# MAEBL Contributes to *Plasmodium* Sporozoite Adhesiveness

**DOI:** 10.3390/ijms23105711

**Published:** 2022-05-20

**Authors:** Mónica Sá, David Mendes Costa, Ana Rafaela Teixeira, Begoña Pérez-Cabezas, Pauline Formaglio, Sylvain Golba, Hélèna Sefiane-Djemaoune, Rogerio Amino, Joana Tavares

**Affiliations:** 1 Host-Parasite Interactions Group, Instituto de Investigação e Inovação em Saúde, Universidade do Porto, 4200-135 Porto, Portugal; monica.sa@ibmc.up.pt (M.S.); david.costa@ibmc.up.pt (D.M.C.); ana.teixeira@ibmc.up.pt (A.R.T.); bperezcabezas@gmail.com (B.P.-C.); 2 Instituto de Biologia Molecular e Celular, Universidade do Porto, 4200-135 Porto, Portugal; 3 Departamento de Ciências Biológicas, Faculdade de Farmácia, Universidade do Porto, 4050-313 Porto, Portugal; 4 Unit of Malaria Infection and Immunity, Institut Pasteur, 75015 Paris, France; pauline.formaglio@pasteur.fr (P.F.); rogerio.amino@pasteur.fr (R.A.); 5 Center for Production and Infection of *Anopheles*, Institut Pasteur, 75015 Paris, France; sylvain.golba@pasteur.fr (S.G.); helena.sefiane-djemaoune@pasteur.fr (H.S.-D.)

**Keywords:** *Plasmodium*, sporozoite, MAEBL, liver, adhesion, genetic complementation, in vivo bioluminescence imaging

## Abstract

The sole currently approved malaria vaccine targets the circumsporozoite protein—the protein that densely coats the surface of sporozoites, the parasite stage deposited in the skin of the mammalian host by infected mosquitoes. However, this vaccine only confers moderate protection against clinical diseases in children, impelling a continuous search for novel candidates. In this work, we studied the importance of the membrane-associated erythrocyte binding-like protein (MAEBL) for infection by *Plasmodium* sporozoites. Using transgenic parasites and live imaging in mice, we show that the absence of MAEBL reduces *Plasmodium berghei* hemolymph sporozoite infectivity to mice. Moreover, we found that *maebl* knockout (*maebl*-) sporozoites display reduced adhesion, including to cultured hepatocytes, which could contribute to the defects in multiple biological processes, such as in gliding motility, hepatocyte wounding, and invasion. The *maebl*- defective phenotypes in mosquito salivary gland and liver infection were reverted by genetic complementation. Using a parasite line expressing a C-terminal myc-tagged MAEBL, we found that MAEBL levels peak in midgut and hemolymph parasites but drop after sporozoite entry into the salivary glands, where the labeling was found to be heterogeneous among sporozoites. MAEBL was found associated, not only with micronemes, but also with the surface of mature sporozoites. Overall, our data provide further insight into the role of MAEBL in sporozoite infectivity and may contribute to the design of future immune interventions.

## 1. Introduction

In 2020 alone, more than 240 million cases of malaria were reported leading to 627,000 deaths. These values represent a substantial increase in the number of malaria case incidence and deaths estimated globally, fueled by the disruptions caused by the COVID-19 pandemic [1]. In 2021, the World Health Organization recommended for the first time a malaria vaccine, RTS,S/AS01, for use in children living in endemic areas with moderate to high transmission [1]. However, this vaccine only confers moderate protection against clinical disease by *Plasmodium falciparum*, the most dangerous human malaria parasite [2]. RTS,S/AS01 targets the circumsporozoite protein (CSP), the protein that densely coats the surface of sporozoites, the parasite stage deposited in the skin of the mammalian host by infected mosquitoes. Sporozoites actively migrate in the skin and invade blood vessels to complete their development in the liver. Inside hepatocytes, a single sporozoite will transform and multiply into thousands of merozoites, the red blood cells infective forms. Sporozoites and ensuing liver stages, called the pre-erythrocytic phase, represent an attractive target for immune interventions [3].

Sera from individuals immunized with radiation-attenuated *P. falciparum* sporozoites, the gold standard malaria vaccine, contain antibodies against multiple pre-erythrocytic antigens highly associated with sporozoite-induced protection [4]. In an attempt to find novel pre-erythrocytic antigens, Peng and colleagues screened a library of *P. falciparum* antigens with sera from volunteers immunized by mosquito bite under chemoprophylaxis with chloroquine [5]. One of the antigens recognized by the sera from most of the individuals was the membrane-associated erythrocyte binding-like protein (MAEBL) [5].

MAEBL is a large type I transmembrane protein composed of two N-terminal cysteine-rich adhesion domains homologous to the apical membrane antigen 1 (AMA-1), named M1 and M2, and a C-terminal cysteine-rich region (C-cys) structurally related with *Plasmodium* Duffy binding-like family of erythrocyte binding proteins [6]. Conserved among *Plasmodium* species [7], MAEBL was initially reported as an erythrocytic-binding protein present in blood-stage parasites [6,8], but was later found to be expressed in sporozoites and late liver stages [9,10,11,12]. Although dispensable for asexual blood-stage growth [13,14,15,16], immunization with MAEBL M2 domain protects animals from dying of a challenge with the lethal *Plasmodium yoelii* YM strain infected red blood cells [17].

MAEBL is required for the colonization of the mosquito salivary glands by sporozoites [13,14,16]. Two main *maebl* transcripts are expressed in sporozoites as a result of the alternative splicing in 3′ exons, encoding a canonical transmembrane and a putative soluble MAEBL isoform [12]. However, only the transmembrane isoform is essential for *P. falciparum* sporozoite infection of salivary glands [16].

In sporozoites, MAEBL is found associated with the micronemes [13,14]. However, immunolabelling studies indicate that its subcellular localization is developmentally regulated during parasite maturation, as it changes from being restricted to the apical pole in immature sporozoites, to covering the surface of mature parasites colocalizing with CSP [11]. In salivary gland sporozoites, the protein was detected both internally and on the parasites surface [11,18]. Nevertheless, antibodies generated against MAEBL domains often recognize multiple bands on western blot analysis of parasite extracts that might hinder conclusions on the localization, particularly when sera reactivity is not evaluated also in a knockout line [18].

While MAEBL was suggested to be dispensable for liver infection by *P. berghei* sporozoites collected from the midgut of mosquitoes [13], MAEBL-deficient *P. falciparum* sporozoites from the hemolymph have been shown to exhibit impaired hepatocyte wounding and invasion capacities along with reduced liver infection of humanized chimeric mice [14]. Indeed, antibodies against MAEBL partially inhibit hepatocyte invasion by sporozoites and/or liver-stage development [5,18], supporting a role for MAEBL in sporozoite infectivity in the mammalian host. In this study, and using the rodent malaria model *P. berghei*, we aimed at understanding the contribution of this protein in the sequence of events that lead to a successful establishment of liver infection by sporozoites.

## 2. Results

### 2.1. Genetic Complementation Reverts the Phenotype of maebl- Parasites in the Mosquito

A *maebl* knockout (*maebl*-) line was generated in a bioluminescent background of *P. berghei*, by replacing the open reading frame (ORF) of *maebl* with the selectable marker *Toxoplasma gondii* dihydrofolate reductase-thymidylate synthase by double-crossover homologous recombination (Figure S1A). Three *maebl*- isogenic lines (*maebl*- B2, B3, and G3) were generated and their genotype was verified by PCR and Southern blot analysis (Figure S1B,C). The absence of *maebl* transcripts was confirmed for the *maebl*- lines by RT-PCR (Figure S1D) and the data presented throughout this work refers to *maebl*- G3 clone. A genetic complementation approach was simultaneously adopted to directly link the defective phenotypes of *maebl*- parasites to the absence of MAEBL. As the *P. falciparum maebl* is transcribed along with the upstream gene as a bicistronic transcript [19], the full-length gene was re-introduced into the original locus of *maebl* together with the human dihydrofolate reductase selectable marker cassette, by a single-crossover homologous recombination event (Figure S1E). A *maebl* complemented isogenic line (*maebl_comp* V3) with the expected genotype was isolated and used in further studies (Figure S1F).

**Figure 1:**
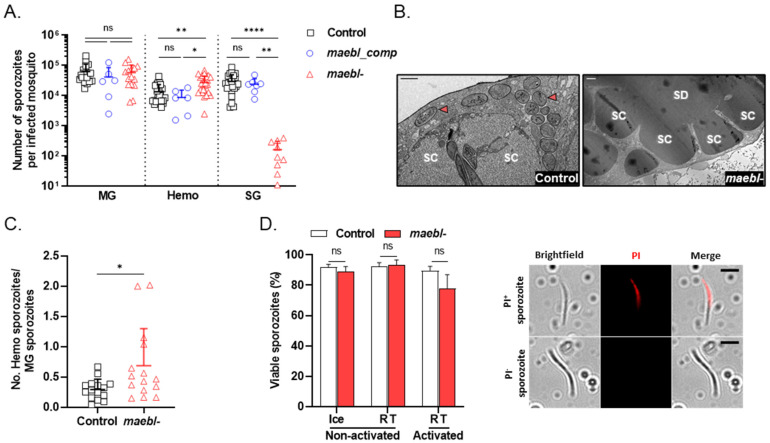
Development of *maebl*- and *maebl_comp* parasites in the mosquito. (**A**) Number of sporozoites in the midgut (MG), hemolymph (Hemo), and salivary glands (SG) of mosquitoes infected with Control, *maebl_comp*, or *maebl*- parasites, on days 18 to 26 post-infection. Symbols represent the counts in independent experiments and bars indicate the mean + SD. Statistical significance was determined using one-way ANOVA (Kruskal–Wallis test with Dunn’s multiple comparisons test). (**B**) Transmission electron micrographs of salivary glands of Control- or *maebl*- -infected mosquitoes, dissected on day 27 post-infection. SC, secretory cavity; SD, salivary duct; red arrows, sporozoites. Scale bar, 1 µm (**left** panel) or 2 µm (**right** panel). (**C**) Ratio of hemolymph (Hemo) to midgut (MG) sporozoites in Control- or *maebl*- infected mosquitoes, between days 18 and 21 post-infection. Symbols represent values of independent experiments and bars indicate the mean + SD. Statistical significance was determined using the Mann–Whitney test. (**D**) Viability of sporozoites. Hemolymph sporozoites were collected from Control- or *maebl*- -infected mosquitoes, on days 18 and 19 post-infection. Sporozoites were activated with DMEM supplemented with 5% FBS at room temperature (activated RT) or incubated with saline phosphate buffer on ice or at room temperature (non-activated Ice and RT, respectively). Propidium iodide (PI) was then added to the parasite suspensions and sporozoites were immediately imaged. **Left**: sporozoites were manually classified as PI+ or PI− sporozoites (dead or viable, respectively). The graphic shows the mean of two independent experiments performed in duplicated + SD. At least 150 sporozoites were analyzed per replicate. Statistical analysis was performed using the unpaired *t*-test. Right: representative images of PI+ or PI− sporozoites. Scale bar, 5 µm. ns, non-significant. * *p* < 0.05; ** *p* < 0.01; **** *p* < 0.0001.

To investigate the development of *maebl*- and *maebl_comp* mutant lines in the vector, mosquitoes were fed on mice infected with control, *maebl*-, or *maebl_comp* parasites. Between days 18 and 26 post-infection, mosquitoes were dissected and the numbers of sporozoites collected from their midguts, hemolymph, and salivary glands were determined. While there were no significant differences between the numbers of midgut sporozoites among all lines, we frequently found higher numbers of *maebl*- sporozoites in the hemolymph, an observation consistent with the inability of these parasites to colonize the salivary glands (Figure 1A) [13]. Transmission electron microscopy (TEM) analysis showed no *maebl*- sporozoites inside salivary glands even when these are collected at a late time point post-infection such as day 27 (Figure 1B), suggesting that the few sporozoites recovered most likely result from contamination with hemolymph. Importantly, we found no differences between the number of control and *maebl_comp* salivary gland sporozoites (Figure 1A), which indicates the genetic complementation rescued sporozoite infectivity to the vector.

In agreement with what was previously reported [13], our data show that *maebl*- sporozoites accumulate in the vector circulatory system (Figure 1A,C). To test whether the accumulation of sporozoites in the hemolymph of infected mosquitoes led to a reduction in parasite viability, we performed a standard propidium iodide (PI) exclusion assay. The percentage of viable *maebl*- hemolymph sporozoites, collected from mosquitoes at day 18/19 post-infection, was not significantly different from that of the control even following activation in the presence of serum (Figure 1D). These results validate the use of *maebl*- hemolymph sporozoites in subsequent experiments.

### 2.2. maebl- Sporozoites Exhibit Decreased Infectivity to Mice

**Figure 2:**
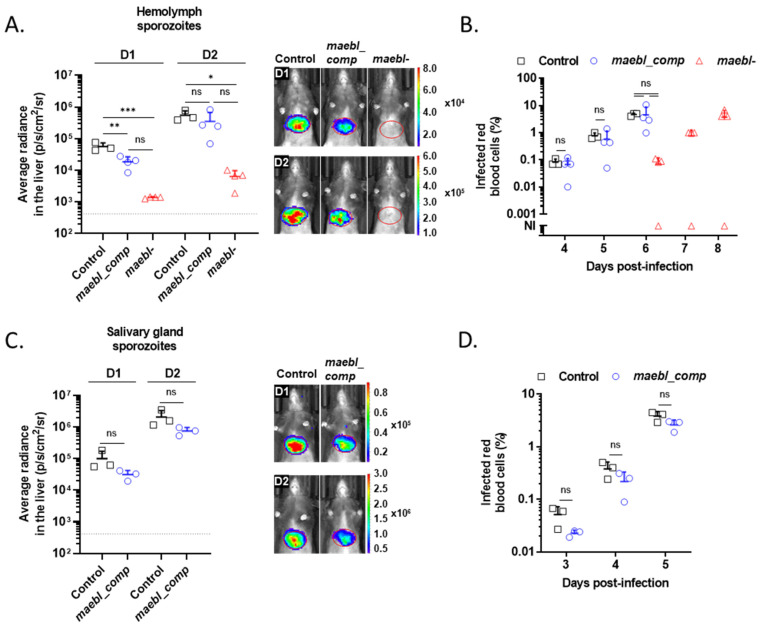
Infectivity of *maebl*- and *maebl_comp* sporozoites to mice. (**A**–**D**) Infectivity of *maebl*- and *maebl_comp* sporozoites to C57BL/6 mice. Mice were injected intravenously with 3.5 × 10^4^ Control, *maebl*- and *maebl_comp* hemolymph sporozoites (panels **A**,**B**) or with 2.5 × 10^4^ Control and *maebl_comp* salivary gland sporozoites (panels **C**,**D**), collected from mosquitoes on days 20 or 21 post-infection. (**A**,**C**) **Left**: parasite burdens in the liver were quantified as average radiance (photons/s/cm^2^/steradian) one (D1) and two (D2) days post-infection. Symbols represent values for individual animals and bars indicate the mean + SD (*n* = 3–4). Dotted line: background level, calculated using non-infected mice. Statistical analysis was performed using one-way ANOVA (Tukey’s multiple comparisons test; panel (**A**) or unpaired *t*-test (panel **C**). **Right**: representative images of infected mice, on D1 and D2 post-infection. (**B**,**D**) Parasitemia of infected mice, determined daily by a Giemsa-stained blood smear. Symbols represent values for individual animals and bars indicate the mean + SD. Statistical analysis was performed using unpaired *t*-test (**B**,**D**) or one-way ANOVA (Tukey’s multiple comparisons test) (**B**). ns, non-significant; * *p* < 0.05; ** *p* < 0.01; *** *p* < 0.001; NI, non-infected.

It has been previously suggested that MAEBL is dispensable for the infection of rat livers by *P. berghei* midgut sporozoites [13]. In contrast, *maebl*- *P. falciparum* sporozoites collected from the mosquito hemolymph showed reduced infectivity to chimeric mice with humanized livers [14]. Therefore, to evaluate whether this phenotype is species-specific [13,14], we assessed the infectivity of *maebl*- and *maebl_comp* hemolymph sporozoites to C57BL/6 mice using in vivo bioluminescence imaging. Mice were inoculated intravenously (i.v.) with control, *maebl*- or *maebl_comp* hemolymph sporozoites, and the bioluminescent signal in the liver was quantified 1- and 2-days post-infection (D1 and D2, respectively). Animals infected with *maebl*- sporozoites showed a reduced liver burden compared to both control and *maebl_comp* at D1 and D2 (Figure 2A). *maebl*- parasites only emerged in the blood of 3 out of 4 mice and after a delay of 2 days comparing with the other lines (Figure 2B). These observations were consistently reproducible, as we frequently observed 1 to 2 days of delay in the prepatent period of mice inoculated i.v. with *maebl*- sporozoites, in several independent experiments (data not shown). Once in the blood, *maebl*- parasites exhibited normal asexual growth kinetics as determined by counting the percentage of infected red blood cells (Figure 2B). Although mice inoculated with *maebl_comp* hemolymph sporozoites displayed lower parasite loads in the liver at D1 compared to control-infected animals (~3.0-fold reduction), the reduction was no longer observed at a later time-point (Figure 2A). In agreement with this observation, no differences were seen in the prepatent periods or in the blood-stage growth of *maebl_comp* and control parasites (Figure 2B).

Genetically complemented *maebl*- sporozoites successfully enter the mosquito salivary glands (Figure 1A). To assess whether *maebl_comp* sporozoites have completed their maturation in the vector and efficiently infect the mammalian host, mice were inoculated i.v. with *maebl_comp* or control sporozoites collected from the mosquito salivary glands. As expected, no differences were observed in the bioluminescent signal between experimental groups at D1 and D2 (Figure 2C), as well as in the parasitemia of animals (Figure 2D). The *maebl*- line was not used in these experiments due to its reduced number of salivary glands-associated sporozoites. Therefore, mice were inoculated i.v. with the few parasites we could collect but no animal ever became blood-stage positive, contrarily to mice infected with similar numbers of control salivary gland sporozoites (Table S1). Altogether, our data demonstrate that in the absence of MAEBL, *P. berghei* hemolymph sporozoites exhibit an impaired ability to infect the liver of mice.

### 2.3. maebl- Hemolymph Sporozoites Present Hampered Invasion and Wounding of Host Cells In Vitro

**Figure 3:**
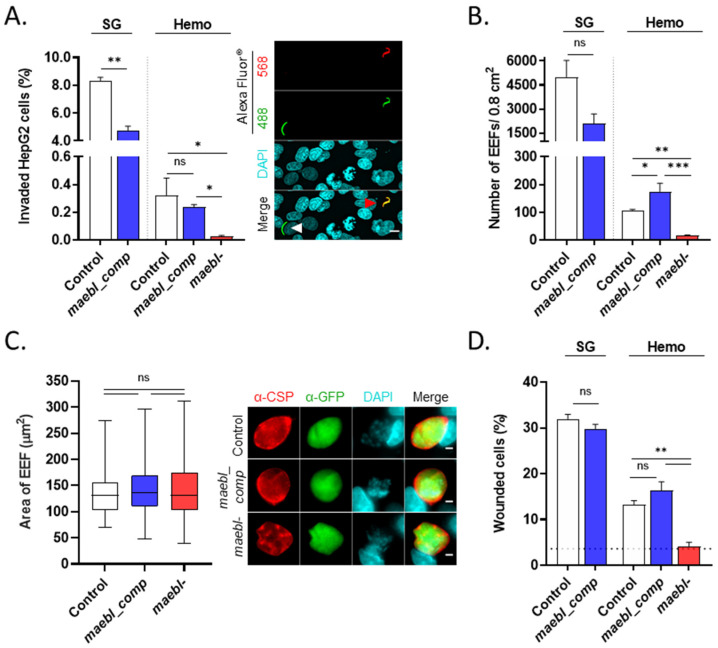
Evaluation of HepG2 cells invasion and cell wounding activity of *maebl*- sporozoites. (**A**–**C**) Invasion of cells by *maebl*- and *maebl_comp* sporozoites and exoerythrocytic forms (EEFs) development. Control, *maebl*- or *maebl_comp* hemolymph (Hemo) or salivary glands (SG) sporozoites, collected from mosquitoes on days 19 to 21 post-infection, were incubated with cells for 2 h (panel **A**) or 48 h (panel **B**,**C**). (**A**) **Left**: percentage of cells containing intracellular sporozoites. Bars represent the mean + SD of experimental replicates. Statistical analysis was performed using unpaired *t*-test (SG) or one-way ANOVA (Tukey’s multiple comparisons test; Hemo). **Right**: representative immunofluorescence images of intracellular (white arrow) and extracellular sporozoites (red arrow). DAPI-stained cell nuclei, cyan. Scale bar, 10 µm. (**B**) Number of EEFs in cells per well. Bars represent the mean + SD of experimental replicates. Values are representative of at least two independent experiments (panel **A**,**B**). Statistical analysis was performed using unpaired *t*-test (SG) or one-way ANOVA (Tukey’s multiple comparisons test; Hemo). (**C**) **Left**: area of EEFs. Box plots showing the median, maximum, minimum, and the 25th and 75th percentiles of the area of individual EEFs. At least 45 EEFs were analyzed per condition. Statistical significance was determined using one-way ANOVA (Kruskal–Wallis test with Dunn’s multiple comparisons test). **Right**: representative immunofluorescence images of EEFs. CSP, red; GFP, green; DAPI-stained nuclei, cyan. Scale bar, 3 µm. (**D**) Cell wounding capacity of Control, *maebl*- and *maebl_comp* Hemo or SG sporozoites collected from mosquitoes on day 19 post-infection. Sporozoites were incubated with cells for 60 min in the presence of propidium iodide (PI). The graph shows the percentage of wounded cells (PI+) assessed by flow cytometry analysis. Bars represent the mean + SD of experimental replicates; values are representative of 3 independent experiments. Horizontal dotted line: percentage of PI+ cells following incubation with medium only. Statistical analysis was performed using unpaired *t*-test (SG) or one-way ANOVA (Tukey’s multiple comparisons test; Hemo). ns, non-significant. * *p* < 0.05; ** *p* < 0.01; *** *p* < 0.001.

Next, we conducted several in vitro experiments to further explore the infectivity of *maebl*- sporozoites to the mammalian host. We started by evaluating sporozoite invasion and liver stage development inside the hepatoma cell line HepG2, using immunofluorescence microscopy. To that end, sporozoites were collected from the hemolymph or salivary glands and incubated with cells for 2 h to evaluate the invasion of host cells. Parasite development was analyzed at 48 h after infection. The percentage of cells with intracellular sporozoites was significantly reduced for the *maebl*- line compared to the control and *maebl_comp* line (Figure 3A), leading to the formation of a lower number of exoerythrocytic forms (EEFs) (Figure 3B). No differences in the size of EEFs were observed among all lines (Figure 3C), suggesting that MAEBL is not required for liver stage development. As expected, *maebl_comp* sporozoites did not show impaired hepatocyte invasion nor exoerythrocytic development (Figure 3A–C).

It has also been demonstrated that cell traversal activity is disrupted in the MAEBL-deficient *P. falciparum* sporozoites [14]. To assess whether this process is also affected in the *P. berghei* knockout line, we performed a standard in vitro cell wounding assay using PI [20]. During traversal, the plasma membrane of host cells is breached, allowing the incorporation of cell-impermeant dyes, such as PI. Thus, sporozoites were allowed to traverse HepG2 cells in the presence of PI for 1 h before quantification of the percentage of wounded cells by flow cytometry analysis. Whereas the percentage of PI+ cells obtained upon incubation with control and *maebl_comp* hemolymph sporozoites was 13.3 ± 0.8% and 16.4 ± 1.8%, respectively, *maebl*- sporozoites induced PI-incorporation levels on host cells close to those of cells incubated with medium alone (4.1 ± 0.9%, Figure 3D and Figure S2). No differences were observed in the percentage of wounded cells by control and *maebl_comp* sporozoites collected from either the hemolymph or the salivary glands (Figure 3D). Altogether, our data indicate that the absence of MAEBL results in a decrease of host cell invasion and wounding by *P. berghei* sporozoites in vitro.

### 2.4. maebl- Hemolymph Sporozoites Glide at Lower Average Speed and Exhibit Defective Attachment

**Figure 4:**
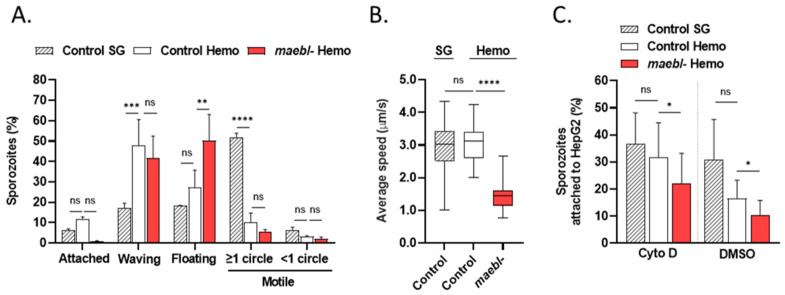
Analysis of *maebl*- sporozoites gliding motility and adhesive properties. (**A**,**B**) Gliding motility of Control salivary gland (SG) and hemolymph (Hemo) sporozoites, and *maebl*- Hemo sporozoites. Sporozoites were allowed to glide in medium supplemented with FBS at 37 °C. Bright-field images were acquired every second for 1 min using an inverted epifluorescence microscope. (**A**) Gliding behaviors of *maebl*- sporozoites. Sporozoites (Control SG *n* = 316, Control Hemo *n* = 296, and *maebl*- Hemo *n* = 839) were classified according to their motility behaviors. Bars indicate the mean of two independent experiments + SD. Statistical analysis was performed using two-way ANOVA (Tukey’s multiple comparisons test). (**B**) Gliding speed of *maebl*- sporozoites. Box plots showing the median, maximum, minimum, and the 25th and 75th percentiles of the average speed of individual sporozoites (Control SG *n* = 20, Control Hemo *n* = 12, and *maebl*- Hemo *n* = 22). Data were pooled from two independent experiments. Statistical analysis was performed using one-way ANOVA (Tukey’s multiple comparisons test). (**C**) Adhesion of Control SG and Hemo sporozoites, and *maebl*- Hemo sporozoites to HepG2 cells. Sporozoites were added on top of cells, in the presence of cytochalasin D (Cyto D) or DMSO. After 30 min incubation at 37 °C, the supernatant was removed to quantify the number of unattached sporozoites. After trypsinization, the number of extracellular parasites (attached sporozoites) was quantified. Quantification of sporozoites was performed by flow cytometry. The high levels of autofluorescence of HepG2 cells precluded the quantification of intracellular sporozoites. The graph shows the mean of three independent experiments performed at least in duplicate + SD. Statistical analysis was performed with repeated measures one-way ANOVA (Tukey‘s multiple comparisons test). ns, non-significant; * *p* < 0.05; ** *p* < 0.01; *** *p* < 0.001; **** *p* < 0.0001.

Since hepatocyte invasion and traversal are two processes dependent on the parasite actin-myosin molecular motor, as well as gliding motility, we next evaluated the motile behaviors of *maebl*- sporozoites in vitro by time-lapse microscopy. Sporozoites were allowed to glide in a polystyrene plate and classified as attached, waving, floating, or motile. As expected, most control sporozoites collected from salivary glands showed vigorous circular gliding contrarily to less mature parasites from the hemolymph of mosquitoes (Figure 4A). Instead, the latter mostly displayed a waving behavior, characterized by the attachment of sporozoites to the surface only by one pole or part of the body (Figure 4A). Although no statistically significant differences were observed between the percentage of control hemolymph and *maebl*- sporozoites that glide at least one complete circle (Figure 4A), mutant parasites showed a significant reduction in their average speed when compared to control sporozoites (Figure 4B).

Strikingly, the *maebl*- line exhibited a significantly higher percentage of floating sporozoites compared to the control (Figure 4A). Based on these observations, we hypothesized that MAEBL could be involved in sporozoite adhesion to the substrate. Indeed, a flawed adhesion could explain the multitude of defects associated with *maebl*- sporozoites (Figure 2A,B, Figure 3 and Figure 4A). To test this hypothesis, we assessed the capacity of *maebl*- sporozoites to attach to HepG2 cells under static conditions. HepG2 cells were incubated with sporozoites for 30 min at 37 °C in the presence or absence of cytochalasin D, an actin polymerization inhibitor, known to impair sporozoite invasion but not adhesion [21], and the numbers of attached and non-attached sporozoites were counted using flow cytometry. Interestingly, we found that mutant sporozoites consistently adhered less than control sporozoites, in the presence of cytochalasin D or the DMSO control only (Figure 4C). Sporozoites collected from the salivary glands tend to adhere more to HepG2 cells than their hemolymph counterparts. However, this trend failed to reach statistical significance (Figure 4C).

### 2.5. Carboxy-Terminal Myc Tagging of MAEBL Does Not Affect Protein Function

We next aimed to investigate in detail the expression and localization of the full-length MAEBL in mature and immature sporozoites. To this end, we engineered a parasite line that expresses a version of MAEBL tagged at the C-terminus end using a functional complementation approach. Briefly, the transfection vector used to generate the *maebl_comp* line was modified as to insert a sequence encoding two myc tag epitopes before the stop codon of *maebl,* and then used to complement the *maebl*- G3 line (Figure S3A). PCR analysis confirmed the correct integration of the construct and the presence of the tagged *maebl* ORF in the genome of three clonal populations (Figure S3B). The clonal population R2 (henceforth named *maebl:myc)* was used throughout this study.

**Figure 5:**
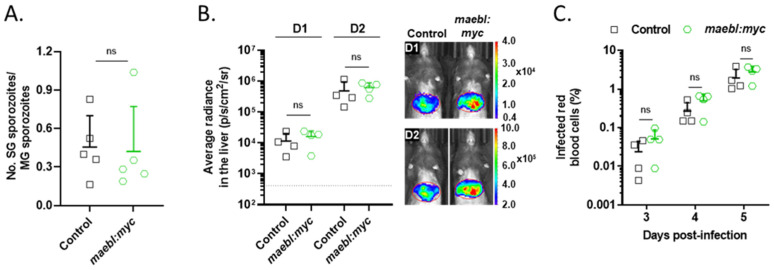
*maebl:myc* sporozoite infectivity to the mosquito and mammalian hosts. (**A**) Ratio of salivary gland (SG) sporozoites to midgut (MG) sporozoites in the Control and *maebl:myc* lines. Sporozoites were collected from mosquitoes on days 17 to 24 post-infection. Symbols represent the counts of independent experiments and bars indicate the mean + SD. Statistical significance was determined using unpaired *t*-test. (**B**) Infectivity of *maebl:myc* sporozoites to C57BL/6 mice by intravenous inoculation with 2.0 × 10^4^ Control or *maebl:myc* salivary gland sporozoites. **Left**: parasite burden in the liver quantified as average radiance (photons/s/cm^2^/steradian) one (D1) and two (D2) days post-infection. Symbols represent values for individual animals and bars indicate the mean + SD (*n* = 4). Dotted line: background level, calculated using non-infected mice. Statistical analysis was performed using unpaired *t*-test. **Right**: Representative images of the infected mice, on D1 and D2 post-infection. (**C**) Parasitemia of the infected mice, determined daily by a Giemsa-stained blood smear. Symbols represent values for individual animals and bars indicate the mean + SD. Statistical analysis was performed using unpaired *t*-test. ns, non-significant.

To assess if the presence of the tag impaired the function of MAEBL, mosquitoes were fed with *maebl:myc* parasites, and the number of sporozoites in the salivary glands was quantified. No differences were observed between the *maebl:myc* and the control line (Figure 5A), indicating that complementation with a C-terminus myc-tagged MAEBL successfully reverted the *maebl*- phenotype in the mosquito. Subsequently, the infectivity of *maebl:myc* sporozoites to the mammalian host was evaluated using in vivo bioluminescence imaging. The parasite load in the liver of C57BL/6 mice inoculated i.v. with *maebl:myc* sporozoites was comparable to control, both at D1 and D2 post-infection, and no delay in the emergence and growth of blood-stage parasites was seen (Figure 5B,C).

### 2.6. Characterization of C-Terminal Myc-Tagged MAEBL Expression and Localization in Sporozoites

**Figure 6:**
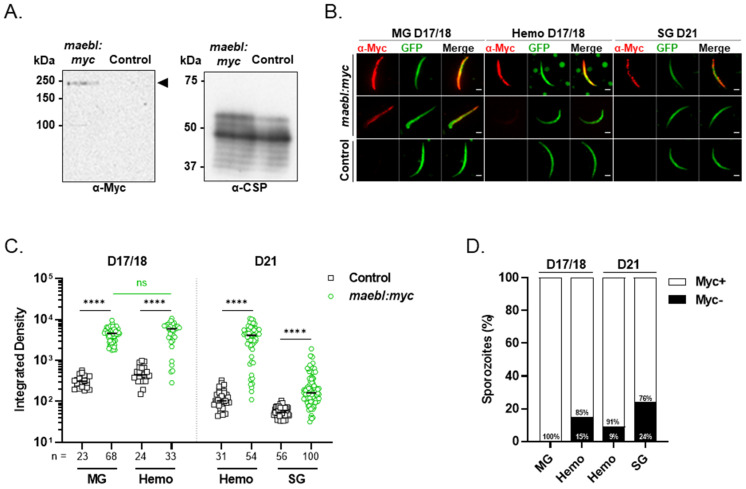
Quantification of myc-tagged MAEBL expression in sporozoites. (**A**) Western blot analysis of myc-tagged MAEBL expression in *maebl:myc* and Control midgut (MG) sporozoite extracts. Denatured lysates of 8.0 × 10^4^ sporozoites were separated on 8% SDS gel and probed with a mouse monoclonal anti-myc tag antibody (clone 4A6). CSP was used as loading control.(**B**–**D**) Quantification of myc-tagged MAEBL expression in sporozoites by immunofluorescence. Sporozoites were collected from the midgut (MG), hemolymph (Hemo) and salivary glands (SG) of mosquitoes infected with Control or *maebl:myc* parasites, on the indicated days. Sporozoites were fixed, permeabilized and labeled anti-myc tag antibody (clone 4A6). (**B**) Representative immunofluorescence images of *maebl:myc* and Control sporozoites stained with anti-myc antibodies visualized in red and the GFP reporter in green. For the representation purpose only, the Smooth filter was applied to the GFP channel. Scale bar, 2 µm. (**C**) Fluorescence intensity of *maebl:myc* sporozoites, quantified as integrated density. Symbols represent individual sporozoites and bars indicate the median value of each population. Statistical significance was determined using the one-way ANOVA (Kruskal–Wallis test with Dunn’s multiple comparisons test). (**D**) Percentage of myc-positive (Myc+) and negative (Myc−) sporozoites. *maebl:myc* sporozoites with a fluorescence intensity superior to the highest value obtained for Control sporozoites were considered positive. ns, non-significant; **** *p* < 0.0001.

A high molecular weight band corresponding to the expected size of the full-length myc-tagged MAEBL (~224 kDa) was detected by Western blot using a monoclonal anti-myc antibody in extracts of *maebl:myc* sporozoites collected from midguts (Figure 6A). Using the same antibody, we next evaluated MAEBL expression in sporozoites by immunofluorescence microscopy. The fluorescence intensity in sporozoites was quantified, and the percentage of positively stained parasites was calculated for parasites presenting an integrated fluorescence value higher than the highest value obtained in control sporozoites. To have a complete overview of the full-length MAEBL expression profile during sporozoite maturation in the vector, sporozoites were collected from different anatomical compartments of the mosquito and at several days post-blood meal. Specifically, *maebl:myc* and control sporozoites were collected as follows: (i) on days 17/18 post-infection, for midgut and hemolymph sporozoites and (ii) on day 21, for hemolymph and salivary gland sporozoites (Figure 6B–D). On days 17/18, all midgut sporozoites analyzed were positive for myc-tagged MAEBL, while only a minor sporozoite population collected from the hemolymph were negative (15%; Figure 6D). However, no significant differences were observed in the signal intensity between both conditions (Figure 6C). When we compare hemolymph parasites collected from mosquitoes a few days later (D21), the percentage of myc-tagged MAEBL-expressing sporozoites reached up to 91% (Figure 6D). On the other hand, salivary gland sporozoites collected at the same day showed not only a lower percentage for the positive population (76%) but also lower signal intensity values compared to hemolymph parasites (Figure 6C,D). This is in agreement with proteomic studies showing that MAEBL is less abundant in salivary gland sporozoites [22,23]. Together, our data indicate that full-length MAEBL is expressed by the large majority of sporozoites during their journey in the vector. Although being abundantly expressed in oocysts, we also found high levels in hemolymph sporozoites, an expected observation considering the role of this protein in sporozoite colonization of the mosquito salivary glands (Figure 1A,B). Importantly, our data clearly shows heterogeneity in full-length MAEBL expression by salivary gland sporozoites.

**Figure 7:**
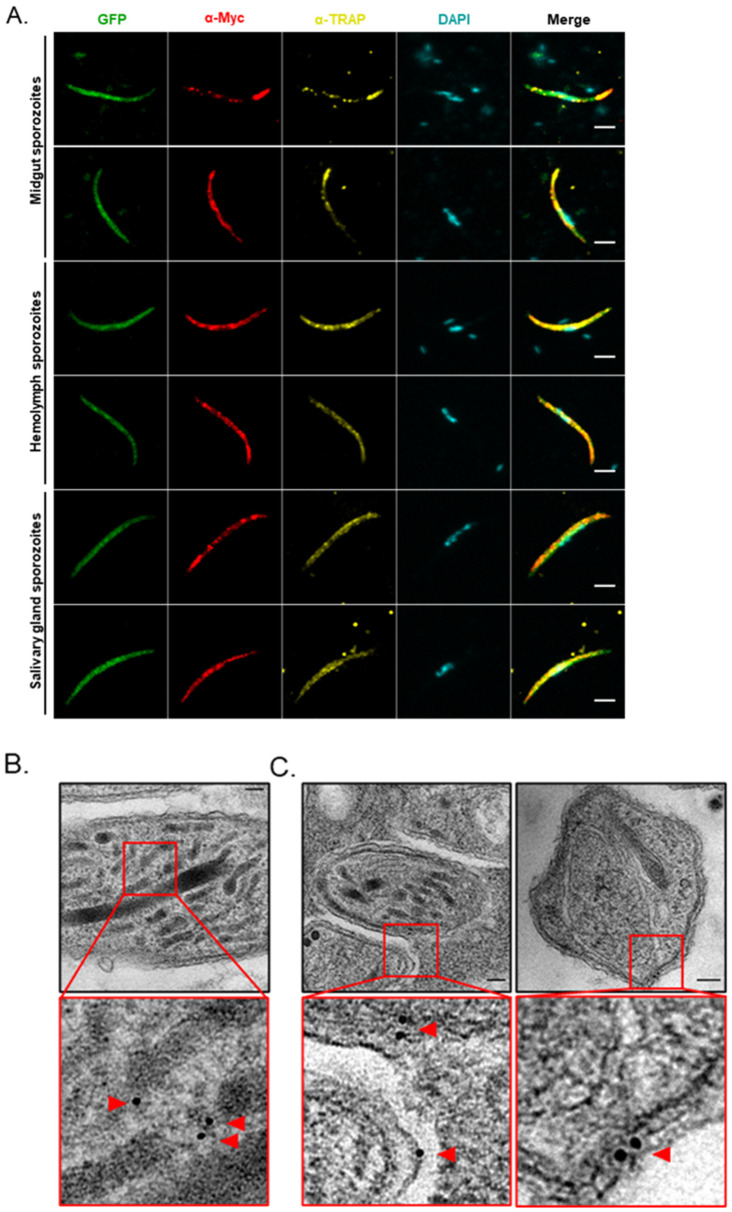
Localization of myc-tagged MAEBL in sporozoites. (**A**) Immunofluorescence analysis of myc-tagged MAEBL distribution by confocal microscopy. *maebl:myc* sporozoites were collected on day 18 (for midgut and hemolymph sporozoites) and day 20 (for salivary glands sporozoites) post-infection, and labeled with a mouse monoclonal anti-myc tag antibody (clone 4A6) and a rabbit polyclonal anti-TRAP repeats antibody. GFP is visualized in green, myc-tagged MAEBL in red, TRAP in yellow, and DAPI-stained nuclei in cyan. For representation purpose only, the Smooth filter was applied in the GFP channel. Images are maximal Z-projections of 7 to 12 contiguous stacks separated by 0.17 to 0.25 µm. Scale bar, 3 µm. (**B**,**C**) Immunoelectron microscopy analysis of salivary glands of *maebl:myc*-infected mosquitoes on day 18 post-infection. Samples were stained with the antibody used in panel (**A**) and secondary antibodies conjugated with 6 nm gold particles. Transmission electron micrographs showing myc-tagged MAEBL in parasite micronemes (panel **B**) and associated with the sporozoite surface (panel **C**). Red arrows, gold particles. Scale bar, 100 nm.

Finally, we evaluated the subcellular localization of myc-tagged MAEBL in sporozoites by confocal microscopy. The staining pattern observed in midgut sporozoites was heterogeneous: whereas some parasites showed strong staining restricted to the apical (and sometimes posterior) pole, in others the signal was found to be more disperse (Figure 7A). In hemolymph sporozoites, myc-tagged MAEBL was frequently distributed throughout the body, and occasionally concentrated towards one (Figure 7A) or both poles (data not shown). Importantly, similar patterns were observed in salivary glands sporozoites (Figure 7A). Partial co-localization with thrombospondin related anonymous protein (TRAP) was frequently observed in midgut and hemolymph sporozoites, confirming the micronemal localization of MAEBL [13,14]. However, colocalization with TRAP in salivary gland sporozoites was not as evident in some sporozoites, as TRAP was frequently found uniformly spread over the sporozoite surface, unlike myc-tagged MAEBL (Figure 7A).

A previous study reported that sera from *P. falciparum* sporozoite-immunized individuals under chloroquine cover recognized MAEBL and antibodies against two MAEBL isoforms blocked the liver stage in vitro, suggesting the protein can reach the surface of the sporozoite and become accessible to antibodies [5]. As our fluorescence microscopy approach required permeabilization of sporozoites before staining as the myc tag is placed at the intracellular portion of the protein, we resorted to immunoelectron microscopy to confirm the subcellular localization of MAEBL in salivary glands sporozoites. Indeed, myc-tagged MAEBL was detected not only associated with micronemes but also to the surface of sporozoites, i.e., near the plasma membrane and/or inner membrane complex (Figure 7B,C).

## 3. Discussion

While MAEBL is dispensable for the asexual growth of parasites in the blood [13,14,15,16], MAEBL-deficient *P. falciparum* sporozoites show impairment in hepatocyte wounding and invasion in vitro, as well as decreased infectivity to humanized chimeric mice [14]. Since previous studies indicate that antibodies against MAEBL can inhibit sporozoites invasion of hepatocytes and/or liver stage development [5,18], its exact contribution to the sequence of events that precedes sporozoite hepatocyte infection is worth exploring. To that end, GFP:luciferase-expressing *P. berghei* parasites were genetically modified to generate several MAEBL mutant lines (Figures S1 and S3) and their phenotype in both the vertebrate and invertebrate hosts analyzed.

Our results indicate that in the absence of MAEBL, *P. berghei* sporozoites show reduced infectivity to the mammalian host (Figure 2A,B), in contrast to previous work [13]. These contradictory findings probably result from the distinct experimental approaches used in both studies, as our experiments were performed with sporozoites collected from the mosquito’s hemolymph instead of parasites collected from the oocysts. Since *maebl*- sporozoites can successfully egress from oocysts but fail to colonize the salivary glands (Figure 1A), we used parasites collected from the mosquito hemocoel, as this transient sporozoite population shows intermediate infectivity to the mammalian host [24]. Noteworthy, we failed every attempt to infect animals with *maebl*- salivary gland-associated sporozoites (Table S1). Considering that we do not find mutant parasites inside salivary glands of mosquitoes (Figure 1B), it is possible that we inoculated mice with hemolymph sporozoites that were collected with the salivary glands. Such low numbers of sporozoites are most likely insufficient to reliably yield productive infections. In addition to the inherently reduced infectivity of hemolymph parasites, *maebl*- sporozoites are 10- to 100-fold less infectious than control parasites, as delays of 1 to 2 days in the prepatent period are frequently observed in mice. Yet, we cannot exclude the possibility of other proteins being up or downregulated in *maebl*- sporozoites, thus, also contributing to the observed phenotype of this line. However, genetic complementation of *maebl- parasites*, rescued the defective phenotype of sporozoites both in the mosquito and the mammalian host (Figure 1A and Figure 2). This confirms that the major impairments associated with *maebl*- sporozoites result from the loss of MAEBL and not from the disarrangement of the *maebl* locus.

On the other hand, the defective phenotype of the *maebl*- line in this study, i.e., loss of cell wounding activity (Figure 3D) and decreased infectivity in vitro (Figure 3A–C) and in vivo (Figure 2A,B), resembles that of MAEBL-deficient *P. falciparum* sporozoites [14], suggesting that the function of this protein is preserved in rodent and human *Plasmodium* infecting species. MAEBL is a conserved protein that predates *Plasmodium* speciation and contains two extracellular N-terminal cysteine-rich domains, named M1 and M2. Each of these domains contains two APPLE domains that are found in bacterial and eukaryotic adhesion molecules [6,25]. Sequence analysis shows that both the number and location of all cysteine residues present in the M1 and M2 domains are evolutionary conserved, suggesting that both regions have significant and similar functions across different *Plasmodium* species [7,10,26]. In blood-stages, it was suggested that MAEBL localizes to the rhoptries and surface of merozoites [7,8] and was shown to possess erythrocyte-binding capacity mainly through the M2 domain [6]. Moreover, it was suggested that MAEBL also participates in the binding of sporozoites to the vector salivary glands [13]. Based on the nature of the M1 and M2 domains and in the multiple defects of *maebl*- sporozoites exhibit in vitro (Figure 3 and Figure 4A,B), we hypothesized that MAEBL contributes to sporozoite adhesion. To test whether MAEBL could be involved in adhesion to host cells, we evaluated the binding of mutant sporozoites to HepG2 using a flow cytometry-based assay. Our data shows that in the absence of MAEBL, sporozoites adhere less to HepG2 cells, both in the presence and absence of cytochalasin D (Figure 4C). These observations, together with the increased percentage of floating *maebl*- sporozoites observed in the gliding assays (Figure 4A), suggest that MAEBL may contribute to the overall adhesion of sporozoites.

As reported previously we did not observe a change in the proportion of motile hemolymph sporozoites in the absence of MAEBL (Figure 4A). Nonetheless, *maebl*- sporozoites glided at a lower average speed compared to controls (Figure 4B), challenging previous conclusions [13]. This could indicate that, for example, the loss of MAEBL may disturb the normal dynamics of discrete adhesion sites formed by sporozoites, as it could participate directly in their formation and/or the turnover, or indirectly, by interfering with the function of other adhesins, such as TRAP or TRAP-related proteins present in such sites [27].

It is likely that the decreased *maebl*- sporozoite infectivity, observed in vitro (Figure 3) and in vivo (Figure 2A,B), results from multiple adhesion-dependent defects. Nevertheless, we cannot exclude a possible role for MAEBL in other steps of hepatocyte invasion, through the interaction with members of the rhoptry neck protein (RON) complex, for example. However, to our knowledge, unlike the structurally related AMA-1, MAEBL was not found associated with RONs [28], suggesting that it acts independently of these proteins during host cell invasion.

We also generated a complemented parasite line expressing a myc-tagged MAEBL (Figure S3). As complementation rescued the phenotype of *maebl*- sporozoites, both in the vector and in the mammalian host (Figure 5), proving it is fully functional, the *maebl:myc* line was used for protein quantification and immunolocalization studies.

In this study, the myc-tag epitope coding sequences were inserted at the C-terminus of *maebl* ORF right before the stop codon. This means that, theoretically, our tagging strategy only allows the detection of the transmembrane isoform (Figure S3C). However, we cannot exclude that due to the alternative splicing other myc-tagged putative soluble and processed forms are also being detected [12]. Nevertheless, our data indicate that MAEBL levels peak in midgut and hemolymph sporozoites (Figure 6C). These observations are in agreement with the crucial function of MAEBL in sporozoite colonization of the vector salivary glands as well as with previous transcriptomic and proteomic studies [22,23,29]. Furthermore, not all salivary gland sporozoites are positive for myc-tagged MAEBL immunolabeling and importantly, protein expression varies within the positive population. Whether MAEBL is expressed de novo in the salivary glands or is carried over from hemolymph parasites remains unknown. Interestingly, recent data from single-cell RNA sequencing reveals extensive transcription heterogeneity among the sporozoite from the same anatomical compartment [29,30]. This could conceivably be an explanation for the fact that we were unable to detect myc-tagged MAEBL in some sporozoites residing in the salivary glands (Figure 6D).

In terms of protein localization, myc-tagged MAEBL frequently colocalized with TRAP (Figure 7A), in agreement with the literature that MAEBL is associated with the micronemes of sporozoites [13,14]. Notably, our data unequivocally indicate that MAEBL was found not only in the micronemes but also associated to the surface of salivary gland sporozoites (Figure 7C), a finding that might be relevant for the design of future immune interventions against *Plasmodium* sporozoites.

## 4. Materials and Methods

### 4.1. Mice, Mosquitoes and Parasites

The *Plasmodium berghei* ANKA strain clone 676cl1 expressing a GFP-Luciferase fusion gene via the *pbef1α* promoter [31], henceforth referred to as control line, was used to generate all mutant lines. C57BL/6, NMRI, and CD1 mice were purchased from Charles River (France) or obtained from the IBMC/i3S animal facility. *Anopheles stephensi* mosquitoes (Sda500 strain) were reared in the Centre for Production and Infection of *Anopheles* (CEPIA) at the Pasteur Institute using standard procedures.

### 4.2. Generation of Transfection Vectors

PCR reactions were performed using a high-fidelity *Taq* DNA polymerase with proofreading activity (Takara Bio, Otsu, Japan) and genomic DNA of control parasites as the template. Primers used for the generation and genotyping of all mutant lines are shown in Table S2. All PCR products were cloned into the pGEM-T Easy vector (Promega, Madison, WI, USA; unless stated otherwise), sequenced (LightRun, Eurofins Genomics, Ebersberg, Germany), and verified against the *P. berghei* genome database (PlasmoDB, http://plasmodb.org/plasmo/, accessed on 22 September 2015) using the Basic Local Alignment Search Tool (BLAST). As a matter of convenience, the intergenic regions upstream and downstream of the *maebl* gene (PBANKA_0901300.2) will be referred to as 5′ and 3′ UTR, respectively.

For the generation of the *maebl* knockout line (*maebl*-), the *maebl* open reading frame (ORF), along with the last 462 bp of the *maebl* 5′UTR, were replaced by the *Toxoplasma gondii* dihydrofolate reductase–thymidylate synthase gene (*TgDHFR/ts*) selectable marker, by a double cross-over homologous recombination event. Part of the *maebl* 5′ (499 bp) and 3′ (493 bp) UTRs were used as homology regions and were amplified using the primer pairs P1/P2 and P3/P4, respectively. The PCR products were subcloned into the plasmid pL0001 (MRA-770; MR4), on each side of the selectable marker, using the restriction sites KpnI/ClaI or EcoRI/BamHI. The final vector was digested with KpnI and BamHI before transfection.

Genetic complementation of *maebl*- clone G3 parasites was achieved by reinserting the wild type coding sequence of *maebl* (*maebl_comp*) or the same gene fused with a sequence encoding two tandem myc tag epitopes (*maebl:myc*), along with the last 462 bp of the *maebl* 5′UTR, into the recombinant locus by a single cross-over homologous recombination event.

The transfection vector containing the untagged *maebl* ORF was obtained as follows. The *maebl* 3′UTR (510 bp) and a 1907 bp DNA fragment corresponding to the first 87 bp of *maebl* ORF, the complete *maebl* 5′UTR and the last 621 nucleotides of the gene upstream of *maebl* (PBANKA_0901400), henceforth referred to as 5′ fragment, were amplified using the primer pairs P5/P6 and P7/P8, respectively. The 3′UTR and the 5′ fragment were inserted into the pGEM-T Easy vector (Promega) or the pCR^®^-TOPO-XL^®^ vector (Invitrogen, Thermo Fisher Scientific, Waltham, MA, USA), respectively, and subcloned into the XhoI/NheI or XmaI/EcoRI sites of a pL0007 vector (MRA-776; MR4). The resulting plasmid was digested with HincII/BsgI to allow the insertion of a 6664 bp fragment that includes the complete coding sequence of *maebl*, flanked by the last 140 bp of the *maebl* 5′UTR and the first 387 bp of the 3′UTR, obtained through the digestion of the *P. berghei* artificial chromosome PbAC02-99h11 (PlasmoGem, Wellcome Sanger Institute, Hinxton, Cambridge, UK) [32,33] with the same restriction enzymes. Finally, the entire DNA sequence ranging from the beginning of the 5′ fragment to the end of the *maebl* 3′UTR (8446 bp) was inserted into a new pL0007 vector digested with HindIII, originating to the final transfection vector pL0007_MAEBLcomp. The correct orientation of the insert was confirmed by EcoRI/HincII digestion. pL0007_MAEBLcomp was linearized with PmeI before transfection.

The transfection vector containing the tagged *maebl* ORF was obtained through modification of the pL0007_MAEBLcomp plasmid by inserting a sequence encoding 2 copies of the myc tag epitope (2× EQKLISEEDL) right before the stop codon. The *maebl* 3′UTR (497 bp) and the last 669 bp of the *maebl* ORF (excluding the stop codon) were amplified using the primer pairs P5/P9 and P10/P11, respectively; the latter primer including the coding sequence of the tag. Both fragments were subcloned into the XhoI/EcoRV and EcoRV/EcoRI sites of a pL0007 vector, originating the plasmid pL0007_MAEBLmyc_3′UTR. The pL0007_MAEBLcomp plasmid was digested with the BstBI restriction enzyme to remove a 6689 bp fragment corresponding to the last 13 bp of the PBANKA_0901400 gene sequence, the entire *maebl* 5′UTR and the first 5475 bp of the *maebl* ORF, henceforth named 5′UTR_ORF fragment. After religation, the resultant plasmid was digested with BstBI/HincII to replace the *maebl* 3′UTR and the final portion of the *maebl* ORF by a myc-tagged version, obtained through the digestion of pL0007_MAEBLmyc_3′UTR with the same restriction enzymes. Finally, the plasmid was digested with BstBI to allow the insertion of the 5′UTR_ORF fragment, originating the final transfection vector. The correct orientation of the insert and the presence of the myc tag epitope coding sequence in the transfection vector were confirmed by DNA sequencing. The plasmid was linearized with PmeI before transfection.

### 4.3. Transfection and Cloning of Mutant Lines

Transfection of schizonts was performed as previously reported [34]. Immediately after electroporation, parasites were injected intravenously into 2 mice (parental populations) and selected with the appropriate drug, starting the day after parasite inoculation. Pyrimethamine (0.07 mg/mL) was given in drinking water, to select *maebl*- parasites. Once parasitemia was above 1%, blood from each animal was transferred into 2 naïve mice (transfer populations) for another round of selection. Selection of *maebl_comp* and *maebl:myc* parasites was performed with WR99210 (Jacobus Pharmaceutical Company, Inc., Princeton, NJ, USA). WR99210 (3.2 mg/mL) was dissolved in dH_2_O 40% (*v*/*v*) ethanol, 3% (*v*/*v*) benzyl alcohol [34], and administrated subcutaneously (16 mg/Kg) for 3 successive days. Once parasitemia was above 1%, blood from each animal was transferred into a naïve mouse (transfer population). The treatment was repeated, starting from the day of infection. Cloning populations were obtained by limiting dilution [35].

### 4.4. Genotypic Analysis of Mutant Parasites by PCR and Southern Blot

Blood from infected mice was collected, filtered through a Plasmodipur filter (EuroProxima, Arnhem, The Netherlands), and lysed with 0.15% (*v*/*v*) saponin. Genomic DNA extraction and purification were done using the QIAamp DNA Blood Mini kit (Qiagen, Hilden, Germany). The integration of the constructs in the expected loci (primers P14/P15 and P7/P18, for the *maebl*- and *maebl*_myc genotyping strategies, respectively), the presence or absence of the *maebl* ORF in the genome of parasites (primers P12/P13), and the presence of the myc tag epitope in the final portion of *maebl* coding sequence (primers P19/P20), were evaluated by PCR using a high-fidelity DNA polymerase (Phusion^®^, New England Biolabs, Ipswich, MA, USA).

For Southern blot analysis, 2.3 to 10 μg of genomic DNA were digested with HindIII/NruI, separated by 0.8% (*w*/*v*) agarose gel electrophoresis, and transferred to a Nytran-N membrane (Amersham Hybond N+, Cytiva, Marlborough, MA, USA). The hybridization probe was obtained by PCR amplification of control DNA, using the primers P1/P2. Labelling of the probe and signal generation were performed using the AlkPhos Direct™ Labeling and Detection System with CDP-Star chemiluminescent detection reagent (Cytiva), respectively.

### 4.5. Evaluation of Gene Expression by Reverse Transcription PCR (RT-PCR)

Total RNA was isolated from midgut sporozoites using the NucleoSpin RNA II kit (Macherey-Nagel, Düren, Germany) and converted into cDNA using the NZY First-Strand cDNA Synthesis kit (NZYTech, Lisbon, Portugal). Detection of the *maebl* cDNA by PCR was done using a high-fidelity DNA polymerase (Phusion^®^, New England Biolabs) and the primers P12/P13. A region of the tubulin beta chain (PBANKA_1206900) was amplified using the primers P16/P17 and used as an internal control. Primer sequences are given in Table S2.

### 4.6. Mosquito Infections and Isolation of Sporozoites

Female mosquitoes were fed on infected NMRI or CD1 mice as described elsewhere [36]. Sporozoites were isolated from mosquitoes 17 to 27 days after the infectious bloodmeal. Midguts and salivary glands were collected into cold Dulbecco’s Phosphate Buffered Saline (DPBS; Gibco, Thermo Fisher Scientific) and disrupted with a pestle immediately before use. Hemolymph sporozoites were isolated by flushing the mosquitos with 10 to 15 µL DPBS and left on ice until use. The total number of sporozoites obtained was determined using a plastic slide with a grid (KOVA^®^ Glasstic^®^ Slides, Kova International, Inc., Garden Grove, CA, USA) and a light microscope.

### 4.7. Sporozoite In Vitro Assays

#### 4.7.1. Viability Assay

Hemolymph sporozoites, collected from mosquitoes on days 18 and 19 post-infection, were incubated for 15 min in DPBS on ice or at room temperature (RT), or at RT after dilution with an equal volume of Dulbecco’s Modification of Eagle’s Medium (DMEM;Lonza, Basel, Switzerland) supplemented with 10% (*v*/*v*) fetal bovine serum (FBS; Biowest, Nuaillé, France). Propidium iodide (PI; 5 µg/mL; Sigma, Merck, Darmstadt, Germany) was added to the suspensions, which were loaded into Ibidi 18-well µ-Slides (Ibidi GmbH, Gräfelfing, Germany), at a density of 5 × 10^3^ to 1 × 10^4^ parasites per well, and immediately imaged using IN Cell Analyzer 2000 (Cytiva). Based on PI incorporation, sporozoites were manually classified as dead or viable (PI+ or PI− sporozoites, respectively), using ImageJ/Fiji analysis software version 1.53f51 (ImageJ, National Institutes of Health, Bethesda, MD, USA). At least 150 sporozoites were analyzed per well and the percentage of viable sporozoites was calculated by dividing the number of PI− sporozoites by the total number of analyzed sporozoites.

#### 4.7.2. Invasion and Liver Stage Development Assays

Host cell invasion and development assays were performed with sporozoites collected from mosquitoes on days 19 to 21 post-infection. The 8-well Lab-Tek chamber slides (Thermo Fisher Scientific) were precoated with 10 µg/cm^2^ of collagen type I from rat tail (Sigma), overnight at 4 °C, if required. HepG2 cells (ATCC) were seeded at 1 × 10^5^ cells per well in DMEM high glucose supplemented with 10% FBS (*v*/*v*) and cultured at 37 °C, 5% CO_2_, for 24 h. Infections of hepatoma cells were performed with 1.4 × 10^4^ to 2.0 × 10^4^ sporozoites in DMEM supplemented with 5% FBS (*v*/*v*), penicillin–streptomycin (100 U/mL; Lonza), for 2 or 48 h at 37 °C, 5% CO_2_, to assess sporozoite invasion and liver stage development, respectively. Preparations were fixed with 4% paraformaldehyde (PFA) (*w*/*v*) in DPBS, for 30 min, and stored at 4 °C until use.

Processing of samples was performed at RT, unless stated otherwise, and the incubation time of all antibodies was 1 h. The percentage of invaded cells was accessed using a double staining strategy [37]. Briefly, samples were blocked with 5% FBS (*v*/*v*) in DPBS, for 30 min, and extracellular sporozoites were labeled with the anti-CSP 3D11 mouse monoclonal antibody (2 μg/mL; MR4) and a goat anti-mouse Alexa Fluor 568 antibody (4 μg/mL; Invitrogen). Following cell permeabilization with 1% (*v*/*v*) Triton X-100 (Sigma), for 4 min, sporozoites were labeled with the same primary antibody in combination with goat anti-mouse Alexa Fluor 488 antibodies (4 μg/mL; Invitrogen™). Cell nuclei were stained with DAPI. Antifade mounting medium [90% (*v*/*v*) glycerol (Alfa Aesar, Thermo Fisher Scientific), 0.5% (*w*/*v*) n-propyl gallate (Sigma), 20 mM Tris-HCl (Sigma), pH 8.0] was added to the preparations and slides were stored at 4 °C until use. Image acquisition was performed using IN Cell Analyzer 2000 (Cytiva). The numbers of sporozoites and HepG2 cell nuclei were determined using the ImageJ/Fiji analysis software (ImageJ, National Institutes of Health) or using an automated counting system, as previously described [38]. The percentage of infected cells was calculated by dividing the total number of intracellular sporozoites by the total number of HepG2 cell nuclei.

To evaluate the development of parasites, slides were blocked with 5% FBS (*v*/*v*) in DPBS, for 30 min, permeabilized with 1% (*v*/*v*) Triton X-100, for 4 min, and labeled with an anti-CSP 3D11 mouse monoclonal antibody (2 μg/mL; MR4) and an anti-GFP rabbit antibody (1:250; MBL, Tokyo, Japan) in combination with secondary antibodies goat anti-mouse Alexa Fluor 568 (4 μg/mL; Invitrogen™) and goat anti-rabbit Alexa Fluor 488 (4 μg/mL; Invitrogen). Nuclei were stained with DAPI. Antifade mounting medium were added to the preparations and slides were stored at 4 °C until use. EEFs were counted by microscopic visualization using a Zeiss Axio Imager Z1 microscope (Carl Zeiss, Oberkochen, Germany) and AxioVision software version 4.9 (Carl Zeiss, Germany), or using an automated counting system, as described previously [38]. To quantify the size of EEFs, images were taken of random EEFs using a Zeiss Axio Imager Z1 microscope (Carl Zeiss, Germany) and the area was manually determined based on the CSP staining, using the ImageJ/Fiji analysis software (ImageJ, National Institutes of Health).

#### 4.7.3. Cell Wounding Assay

The capacity of sporozoites to wound cells was addressed using a standard flow cytometry-based cell-wounding assay [20]. In brief, HepG2 cells were seeded on a 96-well plate at a density of 8 × 10^4^ cells per well in DMEM supplemented with 10% FBS (*v*/*v*) and cultured at for 24 h at 37 °C, 5% CO_2_. The cells were then incubated with ~3 × 10^4^ sporozoites, isolated from mosquitoes on day 19 post-infection, in the presence of 5 μg/mL of PI for 60 min at 37 °C, 5% CO_2_. Uninfected cells, incubated with or without PI, were used as controls. Cells were washed twice with warm DPBS and trypsinized. At least 7.8 × 10^3^ events were analyzed with a FACS Canto II flow cytometer (BD Biosciences, Franklin Lakes, NJ, USA). Data analysis was performed using the FlowJo software version 10.7.1 (FlowJo LLC, Ashland, OR, USA).

#### 4.7.4. Motility Assay

Sporozoites collected from mosquitoes on day 24 post-infection into DPBS were mixed with an equal volume of DMEM supplemented with 10% FBS (*v*/*v*) and transferred into a 384-microwell plate with an optical bottom (Greiner AG, Kremsmünster, Austria). After centrifugation for 5 min at 500× *g*, the plate was placed into the temperature-controlled microscope chamber held at 37 °C. Bright-field images were acquired every second for 1 min, using a widefield inverted Leica DMI6000 (Leica Microsystems GmbH, Wetzlar, Germany) microscope and LAS X software version 3.7.4.23463 (Leica Microsystems GmbH, Germany). Image analysis was performed using the ImageJ/Fiji analysis software (ImageJ, National Institutes of Health). Sporozoites were classified as follows: (i) attached, defined as sporozoites that were completely immobilized at the bottom of the well during the entire video; (ii) waving, defined as sporozoites that were attached only by a portion of the body; (iii) floating or (iv) motile. Motile sporozoites were further subclassified based on the completion or not of a full circle. The average speed was calculated by manually tracking at the apical end on sporozoites that glide at least one complete circle.

#### 4.7.5. Adhesion Assay

Cell adhesion assays were performed with sporozoites collected from mosquitoes on days 19 to 21 post-infection. HepG2 cells were seeded in 96-well plates at a density of 5.0 × 10^4^ to 1.50 × 10^5^ cells per well, in DMEM supplemented with 10% FBS (*v*/*v*) and 1× MEM non-essential amino acids (Sigma), and cultured at 37 °C, 5% CO_2_ until reach confluency. Sporozoites (1.25 × 10^4^), diluted in an equal volume of DMEM supplemented with 10% FBS (*v*/*v*), 1× MEM non-essential amino acids solution (*v*/*v*) and penicillin–streptomycin (200 U/mL; Sigma), were incubated with cells for 30 min at 37 °C, 5% CO_2_ under static conditions, in the presence of 1 µM cytochalasin D (Sigma) or DMSO (Sigma). Following incubation, the supernatant was removed, and cells were washed twice with warm DPBS. Unattached sporozoites, defined as the number of GFP^+^-parasites present in the supernatants, were quantified by flow cytometry. Subsequently, cells were trypsinized and analyzed by flow cytometry, to determine the number of extracellular GFP^+^-sporozoites that were attached to cells. The high levels of autofluorescence of HepG2 cells precluded the quantification of intracellular sporozoites. Data were acquired using a CytoFLEX S flow cytometer (Beckman Coulter, Inc., Brea, CA, USA) and analyzed with the CytExpert version 2.0 (Beckman Coulter, Inc.). The numbers of attached and unattached sporozoites were determined based on sample volume and cell concentration. The percentage of the attached sporozoites was calculated by dividing the number of attached parasites by the total number of sporozoites recovered (attached and unattached).

#### 4.7.6. Quantification and Subcellular Localization of Myc-Tagged MAEBL by Immunofluorescence

Sporozoites were collected from mosquitoes and transferred to an Ibidi 18-well µ-Slides (Ibidi GmbH). Sample processing was performed at RT, unless stated otherwise. Preparations were fixed with 4% PFA (*w*/*v*) in DPBS, for 30 min, permeabilized with 1% (*v*/*v*) Triton X-100, for 4 min, and blocked with 5% FBS (*v*/*v*) in DPBS, for 30 min. Sporozoites were stained with a mouse monoclonal anti-myc tag antibody clone 4A6 (5 μg/mL, Merck), overnight at 4 °C, and with goat anti-mouse Alexa Fluor 568 antibodies (2 μg/mL; Invitrogen™), for 30 min. Between each step (except after blocking), wells were washed five times with DPBS. Antifade mounting medium was added to the preparations and slides were immediately imaged using an inverted epifluorescence Leica DMI6000 microscope (Leica Microsystems) and LAS X software version 3.7.4.23463 (Leica Microsystems). Sporozoite signal intensity was quantified as integrated density (the product of the sporozoite area and the mean grey value), using ImageJ/Fiji software (ImageJ, National Institutes of Health). The background fluorescence was subtracted from the integrated density value for every sporozoite. For each day and condition, control sporozoites were used as negative control. The percentage of myc-positive sporozoites was calculated by dividing the number of *maebl:myc* sporozoites with a fluorescence intensity superior to highest value detected for control sporozoites. For illustrative purpose only, the Smooth filter of the ImageJ/Fiji software was applied to the GFP channel in the representative sporozoite images.

To study of the subcellular localization of myc-tagged MAEBL, *maebl:myc* sporozoites were stained with anti-myc tag antibodies as described above. Additionally, sporozoites were probed with a rabbit polyclonal anti-TRAP repeats antibody (1:10,000), overnight at 4 °C, stained with goat Alexa Fluor^®^ 647 anti-rabbit IgG antibodies (2 μg/mL; Life Technologies, Thermo Fisher Scientific), for 30 min at RT, and incubated with DAPI (1:5000), for 10 min at RT. The wells were washed with DPBS between each step. Finally, preparations were mounted with antifade solution and immediately imaged. Images were acquired using an inverted microscope Leica TCS SP5 II (Leica Microsystems) and LAS AF software version 2.6.3.8173 (Leica Microsystems), and processed using ImageJ/Fiji software (ImageJ, National Institutes of Health) by projecting the maximum intensity of 7 to 12 contiguous z-stacks, separated by 0.17 to 0.25 µm. For illustrative purpose only, the Smooth filter of the ImageJ/Fiji software was applied to the GFP channel in the representative images of sporozoites.

#### 4.7.7. Transmission Electron Microscopy

Infected salivary glands were collected 27 days post-infections and fixed in 2.5% (*w*/*v*) glutaraldehyde (Electron Microscopy Sciences, Hatfield, PA, USA) and 2% (*w*/*v*) formaldehyde (Electron Microscopy Sciences) in 0.1 M sodium cacodylate buffer (pH 7.4) for 24 h. Samples were washed in buffer, postfixed with 2% (*w*/*v*) osmium tetroxide (Electron Microscopy Sciences) in 0.1 M sodium cacodylate buffer (pH 7.4) for 2 h, washed with water and incubated with 1% (*w*/*v*) uranyl acetate (Electron Microscopy Sciences) overnight. Subsequently, samples were dehydrated with ethanol and embedded in EPON resin (Electron Microscopy sciences). Ultrathin sections of 50 nm thickness were cut using an ultramicrotome (RMC PowerTome XL, Boeckeler Instruments, Inc., Tucson, AZ, USA), mounted on mesh copper grids, and stained with uranyl acetate substitute (Electron Microscopy sciences) and lead citrate (Electron Microscopy sciences) for 5 min each. Samples were visualized using a JEOL JEM 1400 transmission electron microscope (JEOL Ltd., Tokyo, Japan). Images were digitally recorded using a CCD digital camera Orius 1100 W (Japan) and analyzed using ImageJ/Fiji software (ImageJ, National Institutes of Health).

For the detection of myc-tagged MAEBL in salivary gland sporozoites by immunoelectron microscopy, salivary glands of mosquitoes were collected on day 18 post-infection, fixed in 0.05% (*w*/*v*) glutaraldehyde, 2% (*w*/*v*) PFA, 4% (*w*/*v*) sucrose in 0.1 M PBS, for 1 h, and washed with PBS. Samples were sequentially postfixed with 2% (*w*/*v*) osmium tetroxide in 0.1 M sodium cacodylate buffer (pH 7.4) for 1 h, washed with water, incubated with 1% (*w*/*v*) uranyl acetate for 45 min, dehydrated with ethanol and embedded in EPON resin. Ultrathin sections of 60 nm thickness were mounted on mesh nickel grids and processed as follows. Sections washed with Tris-buffered saline (TBS), incubated with 14.4% (*w*/*v*) sodium metaperiodate (Merck) for 1 h, washed with TBS, immersed with 10–20 mM glycine (±0.15%; *w*/*v*) for 5 min, blocked with 2% (*w*/*v*) BSA (AURION BSA-c™, Wageningen, The Netherlands) in TBS for 30 min and incubated with a mouse monoclonal anti-myc tag antibody clone 4A6 (100 μg/mL, Merck) in 2% (*w*/*v*) BSA 3% (*w*/*v*) NaCl in TBS, overnight at 4 °C. Sections were washed with 0.1% (*w*/*v*) BSA in TBS, incubated with 1% (*w*/*v*) BSA in TBS for 20 min, and then with goat anti-mouse secondary antibodies conjugated to 6 nm gold particles (1:20, Abcam, Cambridge, UK) diluted in 1% (*w*/*v*) BSA in TBS, for 1 h. Finally, sections were washed with TBS, post-fixed in 1% (*w*/*v*) glutaraldehyde in TBS for 5 min, washed with water and stained with uranyl acetate substitute and lead citrate for 1 min each. Samples were viewed using a JEOL JEM 1400 transmission electron microscope (JEOL Ltd.). Images were digitally recorded using a CCD digital camera Orius 1100 W (Japan) and analyzed using ImageJ/Fiji software (ImageJ, National Institutes of Health).

#### 4.7.8. Western Blot Analysis

Sporozoites were collected from the midgut of mosquitos on days 17 and 18, mechanically liberated from oocysts, filtered using a 35 μm cell strainer cap (Falcon), and stored at −80 °C until use. Lysates of 8.0 × 10^4^ sporozoites supplemented with cOmplete™, EDTA-free Protease Inhibitor Cocktail (Roche, Basel, Switzerland) were denatured in 2× Laemmli buffer (0.25 M Tris-HCl, pH 6.8, 5% SDS, 20% glycerol 0.02% bromophenol blue, 2.5% β-Mercaptoethanol), for 10 min at 95 °C. Samples were diluted to 1× Laemmli buffer and separated on an 8% (*w*/*v*) acrylamide gel by SDS-PAGE. Proteins were allowed to transfer to a PVDF membrane using a wet transfer system, for 16 h at 20 V, in 1× Towbin buffer [25 mM Tris, 192 mM Glycine, 20% (*v*/*v*) methanol] with 0.025% (*w*/*v*) SDS. After transfer, the membrane was rinsed with PBS and blocked with 5% (*w*/*v*) skim milk, 0.1% Tween 20 (Sigma) in PBS, for at least 1 h at RT. Incubations with a mouse monoclonal anti-myc tag antibody clone 4A6 (5 µg/mL, Merck) or with anti-CSP 3D11 mouse monoclonal antibody (0.3 μg/mL; MR4), diluted in blocking solution, were performed overnight at 4 °C or for 1 h at RT, respectively. The membranes were washed, probed with horseradish peroxidase-conjugated goat anti-mouse secondary antibodies (1:5000; SouthernBiotech, Birmingham, AL, USA) diluted in blocking buffer, for 1 h at RT, and washed again. Signal detection was performed using SuperSignal West Pico Chemiluminescent Substrate (Thermo Scientific, Thermo Fisher Scientific) and Amersham Hyperfilm ECL (Cytiva). Films were revealed using the Fujifilm FPM-100A film processor (Fujifilm, Tokyo, Japan).

### 4.8. Sporozoite In Vivo Assays

To assess mutant sporozoite infectivity and liver-stage development in vivo, C57BL/6 mice were injected i.v. with hemolymph or salivary gland sporozoites, isolated from mosquitoes at day 20 to 25 post-infection.

#### 4.8.1. Bioluminescence Imaging

Bioluminescence imaging was performed as previously described [39], using the IVIS Lumina LT System (PerkinElmer, Inc., Waltham, MA, USA). Mice were imaged 1- and 2-days post-infection to quantify parasite loads in the liver. Before image acquisition, the ventral fur of mice was depilated with an appropriate clipper. Animals were anesthetized with isoflurane and injected subcutaneously with 2.4 mg of D-luciferin potassium salt (PerkinElmer, Inc.) dissolved in DPBS, 5 min before image acquisition. A non-infected mouse was routinely imaged in parallel to evaluate background noise signal. Quantitative analysis in the anatomical region of interest (ROI) encompassing the liver was performed using the Living Image software version 4.4 (PerkinElmer, Inc.), as previously described [39].

#### 4.8.2. Parasitemia

Parasitemia was assessed daily by analysis of Giemsa-stained thin blood smears, starting on day 3 or 4 post-inoculation. The prepatent period was defined as the number of days until mice reached 0.1% parasitemia.

### 4.9. Statistical Analysis

Statistical analyses were performed using the GraphPad Prism Software (version 9.3.0). Statistical significance: *p* < 0.05 (*), *p* < 0.01 (**), *p* < 0.001 (***), *p* < 0.0001 (****).

## 5. Conclusions

In conclusion, we show that MAEBL is required for the optimal adhesiveness of sporozoites. Indeed, we propose that a flawed adhesion is likely to impair subsequent processes such as gliding motility, hepatocyte traversal, and invasion, and ultimately lead to decreased infectivity in vivo. Our work contributes to a better understanding of the role MAEBL plays in sporozoite infectivity to the mammalian host.

## Data Availability

The original contributions presented in the study are included in the article, further inquiries can be directed to the corresponding authors.

## Supplementary Materials

The following supporting information can be downloaded at: https://www.mdpi.com/article/10.3390/ijms23105711/s1.
