# Supplementary material for: MAEBL Contributes to Plasmodium Sporozoite Adhesiveness
Source: Int J Mol Sci. 2022 May 20;23(10):5711. doi: 10.3390/ijms23105711 (PMC9146008; doi:10.3390/ijms23105711)
Supplement: Supplementary file 1 [file ijms-23-05711-s001.zip › ijms-1707243-supplementary.pdf]

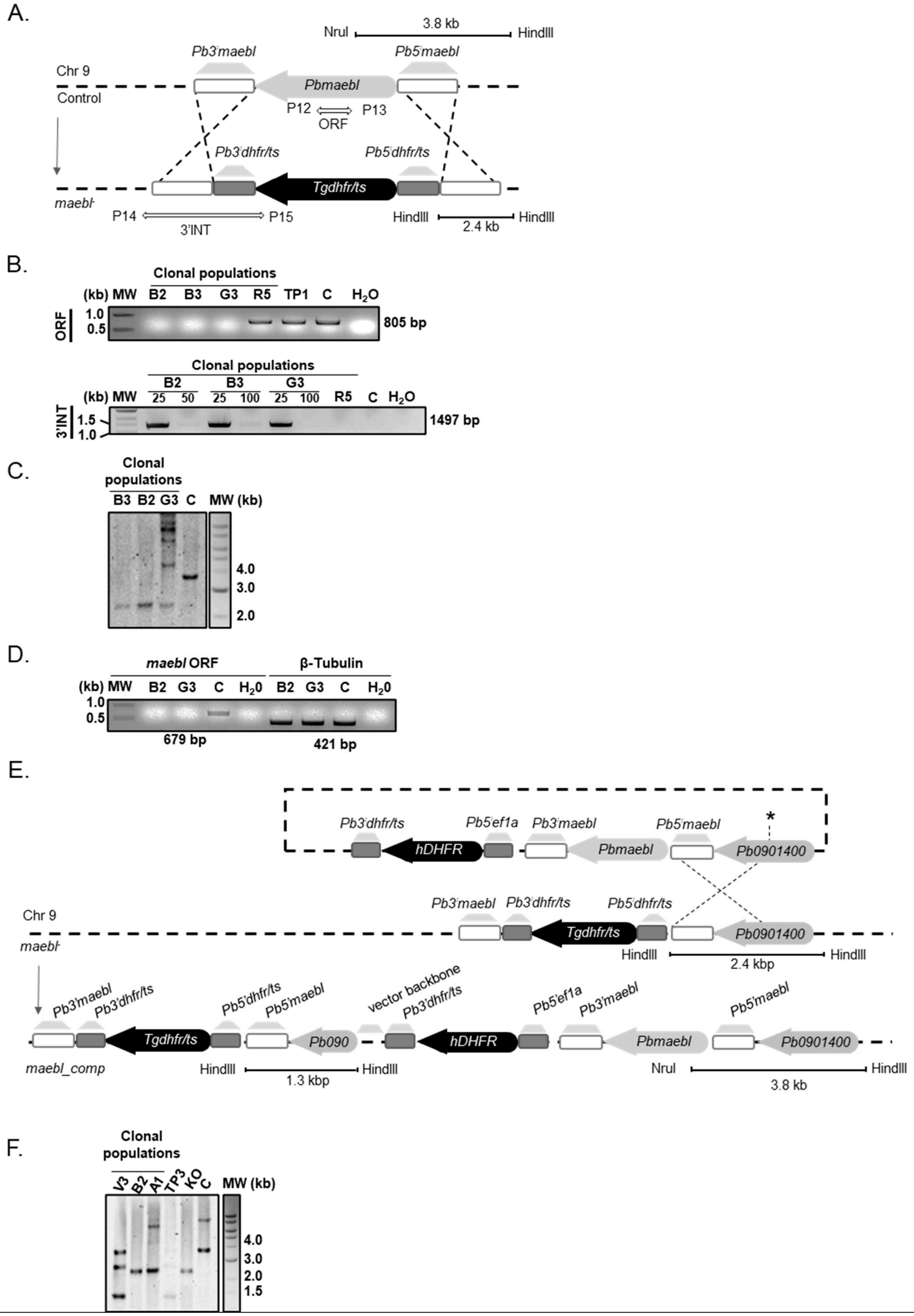
A.
NruI
3.8 kb
HindIII
Pb3'maebl
Pb5'maebl
Chr 9
Control
Pbmaebl
P12
P13
ORF
Pb3'dhfr/ts
Pb5'dhfr/ts
Tgdhfr/ts
maebl-
P14
P15
3'INT
HindIII
2.4 kb
HindIII
B.
Clonal populations
(kb) MW B2 B3 G3 R5 TP1 C H2O
ORF
1.0
0.5
805 bp
Clonal populations
B2 B3 G3
(kb) MW 25 50 25 100 25 100 R5 C H2O
3'INT
1.5
1.0
1497 bp
C.
Clonal populations
B3 B2 G3 C MW (kb)
4.0
3.0
2.0
D.
maebl ORF
β-Tubulin
(kb) MW B2 G3 C H20 B2 G3 C H20
1.0
0.5
679 bp
421 bp
E.
Pb3'dhfr/ts
Pb5'ef1a
Pb3'maebl
Pb5'maebl
*
hDHFR
Pbmaebl
Pb0901400
Chr 9
Pb3'maebl
Pb3'dhfr/ts
Pb5'dhfr/ts
Tgdhfr/ts
Pb0901400
maebl-
HindIII
2.4 kbp
HindIII
Pb3'maebl
Pb3'dhfr/ts
Pb5'dhfr/ts
Pb5'maebl
vector backbone
Pb3'dhfr/ts
Pb5'ef1a
Pb3'maebl
Pb5'maebl
Tgdhfr/ts
Pb090
hDHFR
Pbmaebl
Pb0901400
maebl_comp
HindIII
1.3 kbp
HindIII
NruI
3.8 kb
HindIII
F.
Clonal populations
V3 B2 A1 TP3 KO C MW (kb)
4.0
3.0
2.0
1.5

Figure S1. Generation of *maebl-* and *maebl_comp* parasites. (**A**) Schematic representation of the strategy for the deletion of *maebl* (*maebl-*) in GFP-luciferase-expressing *Plasmodium berghei* ANKA parasites (Control) [31], by double-crossover homologous recombination. The *maebl* 5′ and part of the 3′ intergenic regions (Pb5′ and *3′maebl*) were used as homology arms and cloned on each side of the selectable marker *Toxoplasma gondii dihydrofolate* reductase-thymidylate synthase gene (*TgDHFR/ts*). The location of primers used for genotyping by PCR, the Southern blot strategy, and expected fragment sizes are also represented. (**B**) PCR analysis of clonal populations B2, B3, G3, and R5. The absence of the *maebl* open reading frame (ORF) and the integration (3′INT) of the targeting construct into the genome of mutant parasites was assessed. The 3′INT PCR was performed using different amounts of genomic DNA (gDNA; 25 ng, 50 ng and 100 ng). gDNAs of Control (C) and *maebl-* transfer population 1 (TP1) parasites were used as controls. The expected sizes of the PCR fragments are shown alongside the gels. (**C**) Southern blot analysis of three *maebl-* clones (B2, B3, and G3) and Control parasites. (**D**) RT-PCR analysis of *maebl* expression. cDNAs from *maebl-* clones B2 and G3, and Control (C) midgut sporozoites were used. The *maebl* ORF PCR was performed using the primer pair P12/P13 (depicted in panel A). β-Tubulin was used as a reference gene. (**E**) Schematic representation of the genetic complementation strategy used to generate *maebl_comp* parasites. The targeting vector containing the complete *maebl* ORF (Pbmaebl) flanked by the 5′ and part of the 3′ intergenic regions (Pb5′ and *3′maebl*), the human dihydrofolate reductase (hDHFR) cassette as a selectable marker, and the final portion of the upstream gene (Pb0901400; containing the vector linearization site indicated by the asterisk) was integrated into the *maebl-* G3 recombinant locus by a single-crossover recombination event. The Southern blot strategy and expected fragment sizes are also represented. (**F**) Southern blot analysis of three *maebl_comp* clonal populations (V3, B2, and A1). gDNAs of *maebl_comp* transfer population 3 (TP3), *maebl-* G3 (KO) and Control (C) parasites were used as controls. bp, base pairs; kb, kilobase pairs; MW, molecular weight.

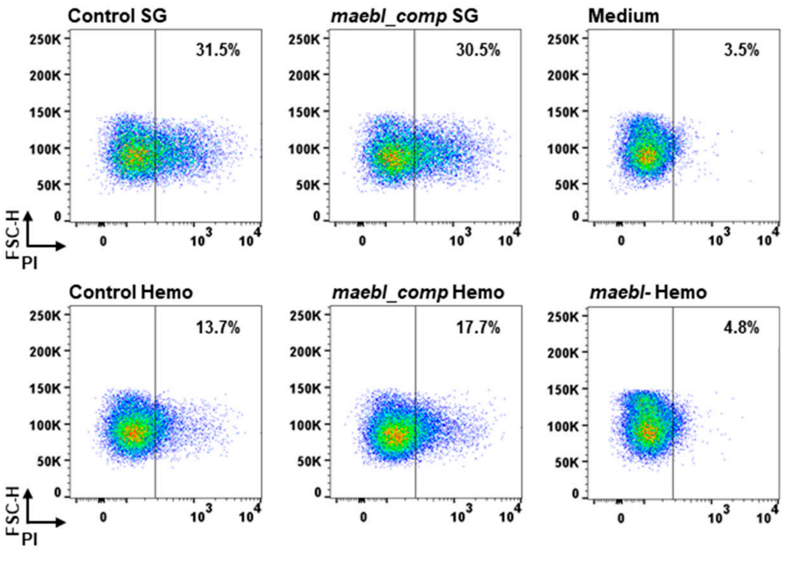

Figure S2. Cell wounding activity *in vitro* of *maebl-* and *maebl_comp* sporozoites. Flow cytometry dot-plots showing the percentage of propidium iodide-positive cells following 60 min incubation with Control, *maebl_comp* or *maebl-* sporozoites, isolated from salivary glands (SG) and hemolymph (Hemo) of mosquitoes on day 19 post-infection, or with medium only (Medium).

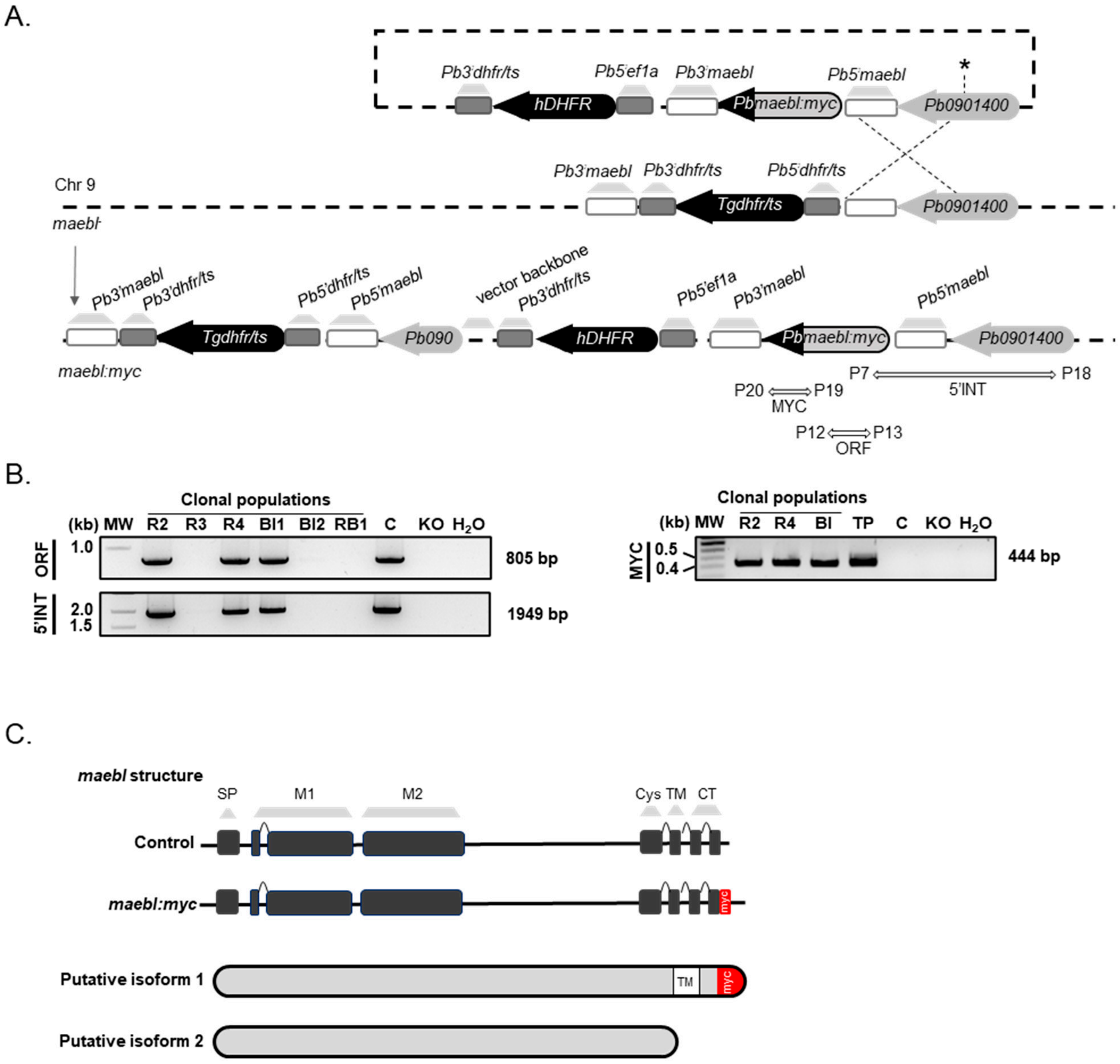

Figure S3. Generation of parasites expressing myc tagged-MAEBL. **(A)** Schematic representation of the complementation strategy used to generate *maebl:myc* parasites. The targeting vector containing a sequence encoding MAEBL fused with two tandem myc epitope tags at C-terminal (*Pbmaebl:myc*), the 5′ and part of the 3′ intergenic regions (*Pb5′* and *3′maebl*), the selectable marker human dihydrofolate reductase (*hDHFR*), and the final portion of the upstream gene (Pb0901400; containing the vector linearization site indicated by the asterisk) was integrated into the *maebl-* clone G3 recombinant locus by a single-crossover recombination event. **(B)** PCR analysis of clonal populations R2, R3, R4, Bl1, Bl2 and RB1. The presence of the *maebl* ORF and the myc tag (MYC), as well as the correct integration (5′INT) of the targeting construct into the genome of transfected parasites, was assessed. gDNAs of Control (C), *maebl-* G3 (KO), and *maebl:myc* transfer population 1 (TP1) parasites were used as controls. The expected sizes of PCR fragments are shown alongside the gels. bp, base pairs; kb, kilobase pairs; MW, molecular weight. **(C)** Schematic representation of *maebl* structure in Control and *maebl:myc* parasites and putative MAEBL isoforms. The *maebl* structure scheme was performed based on [18]; dark gray boxes represent regions encoding for the signal peptide (SP), the M1 and M2 domains, the carboxyl cysteine-rich region (Cys), the transmembrane domain (TM) and cytoplasmic tail domain (CT), ∧ represent

introns. The transmembrane myc-tagged MAEBL putative isoform (1), and a soluble isoform lacking the transmembrane domain (2) are also represented.

Table S1. *maebl-* salivary gland-associated sporozoites infectivity to the mammalian host. C57BL/6 mice were injected intravenously with 340 to 600 Control or *maebl-* salivary glands sporozoites, isolated from mosquitoes at days 19-21 post-infection. In one experiment, 3 animals were inoculated with 1.000 *maebl-* salivary glands sporozoites collected from mosquitoes at day 24/25 post-infection. The presence or absence of blood-stage parasites in the inoculated animals was determined daily by a Giemsa-stained blood smear. i.v., intravenously.

Parasite line	No. of sporozoites injected i.v.	No. of infected animals/No. inoculated animals
Control	340-600	8/8
maebl-	340-600	0/4†
maebl-	1,000	0/3†

†No parasites were seen in the blood of animals within at least 14 days post-infection.

Table S2. Oligonucleotides used in this study.

Primer name		Sequence 5' → 3'
P1	KpnI_5UTR_MAEBL_F	TTGGTACCGCAAAGAACAACATGCATAC
P2	ClaI_5UTR_MAEBL_R	GCATCGATGAAAGACACGAAACACAAG
P3	EcoRI_3UTR_MAEBL_F	GGGAATTCTCGCCCCAATTATATTACC
P4	BamHI_3UTR_MAEBL_R	TTGGATCCGCTAAGAAAGCTTGCCATAC
P5	3'MAEBL_PAC_XhoI_R	GACTCGAGCTAAGAAAGCTTGCCATAC
P6	3'MAEBL_PAC_NheI_F	TAGCTAGCCGAACAAATATAGGAATCGC
P7	5'MAEBL_PAC_XmaI_F	AACCCGGGTTCTTGTGGGTTATCTATGC
P8	5'MAEBL_PAC_EcoRI_HindIII_R	ACGAATTCAAGCTTAAGAGGGCTTCAGTCC
P9	3'MAEBL_TAG_EcoRV_F	TCGATATCGAATCGCCCCAATTATATTAC
P10	MAEBL_ORF_EcoRI_F	TCGAATTCAAGTTGTTCGAATGATGAAAG
P11	MAEBL_ORF_TAG_EcoRV_R	CCGATATCCTAtaagtcttcctcacttattaacttctgttccagatcctcttctgagatga gtttttgttcTATTTGTTCGTTAGC
P12	MAEBL_ORF_R	ACATGGCCCGATACAATGAG
P13	MAEBL_ORF_F	GGGCATAGATAACCCACAAG
P14	MAEBL_locus_3'	CAACCGTCGAAGGCATAAATTAG
P15	TgDHFR/TS_ORF	CCCATTGTGAACATCCTCAAC
P16	Tubulin β-chain_F	TGGAGCAGGAAATAACTGGG
P17	Tubulin β-chain_R	ACCTGACATAGCGGCTGAAA
P18	MAEBL_5locus_int	ATATCTTCGTCAAGCGAATC
P19	MAEBL_ORFend_F	GGCGGGAATAATAATGTCCATC
P20	TAG_Myc_R	CTTATTAACTTCTGTTCCAGATCC

Restriction sites are underlined.
Letters is red represent a stop codon.
Lower case letters represent a sequence encoding 2x myc tag epitope EQKLISEEDL.
